
JOURNAL INFORMATION
==============================
NLM Title Abbreviation: Innov Surg Sci
Journal ID: Innov Surg Sci
NLM Journal ID: 101708165
Journal ID: iss

Innovative Surgical Sciences

EISSN: 2364-7485
Publisher: De Gruyter

ARTICLE INFORMATION
==============================
PMCID: PMC10216885
DOI: 10.1515/iss-2023-9003
Article ID: iss-2023-9003
Article version: 1
Subjects: Article

Abstract DGNC

Publication date: 2023 Mar
Electronic publication date: 2023 Mar 13
Volume: 8
Issue: Suppl1
First page: 19
Last page: 30
Copyright: © the Author(s), published by De Gruyter
Copyright year: 2023
Copyright holder: the Author(s), published by De Gruyter
License: This work is licensed under the Creative Commons Attribution 4.0 International License.
License URL: https://creativecommons.org/licenses/by/4.0/

Page count: 12

==============================

**Image-guided screw placement in the upper cervical spine: analysis of accuracy and safety of different fluoroscopic techniques**

ID: 67

**Kategorie: DGNC - Intraoperative Bildgebung und Robotik an der Wirbelsäule**

Ramazan Jabbarli[1], Marvin Darkwah Oppong[1], Laurèl Rauschenbach[1], Oliver Gembruch[1], Philipp Dammann[1], Ulrich Sure[1], Karsten H. Wrede[1]

[1]*Universitätsklinikum Essen, Klinik für Neurochirurgie und Wirbelsäulenchirurgie, Essen, Deutschland*

**Background:**

Due to the complex anatomy of the upper cervical spine, screw placement in C1 and C2 might become challenging with a high risk of intraoperative complications. In this study, we assessed the accuracy and safety of C1 and C2 screw placement using one of three techniques: standard biplanar fluoroscopy (SBF) vs. navigated screw placement with 3D fluoroscopy (n3DF) or robotic cone-beam CT (nRCC) guidance.

**Materials and methods:**

The study group comprised all consecutive cases with image-guided posterior screw placement in C1 and/or C2 treated in our institution between 2009 and 2022. Preoperative CT scans were analyzed regarding anatomic characteristics of the vertebrae, as well as an optimal screw position (with longest possible intraosseous course) using 3D reconstruction with a navigation software for all cases. The accuracy of screw placement was assessed in the postoperative CT scans. The rates of screw-related complications, intraoperative screw repositions, and screw revision surgeries were recorded.

**Results:**

By June 2022, 80 image-guided surgeries for screw placement in the upper cervical spine were included (n=41/28/11 for SBF/n3DF/nRCC; mean age: 57.9 years; 60% males). There were no cases of new neurologic deficits or vertebral artery injury related to screw malposition. Regarding the results of C1 screws (n=93), navigated screw placement showed higher accordance with the modulated optimal screw position (90.7% vs 83.3%, p<0.0001), lower extent of cortical breaching (0.4 vs 1.4 mm, p=0.003), and no screw revision surgery (0% vs 9.7%, p=0.035) as compared to SBF-guided technique. In the C2 screw subgroup (n=97), navigated screws were also superior with regard to the optimal screw position (84.6% vs 50%, p<0.0001) and cortical breaching rate (12.8% vs 34.5%, p=0.019) compared to the non-navigated screws. When comparing both techniques of navigation-guided screw placement, there was a lower rate of cortical breach (C1: 18.8% vs 34.8%, p=0.347; C2: 7.1% vs 32%, p=0.119), narrowing of the vertebral canal (C1: 0% vs 4.3%. p=1.000; C2: 0% vs 4.0%, p=1.000), and intraoperative screw revision (C1: 0% vs 4.3%, p=1.000; C2: 0% vs 8.0%, p=0.528) in the nRCC- than in n3DF-navigated cases.

Open Access. © 2023 The Author(s), published by De Gruyter. This work is licensed under the Creative Commons Attribution 4.0 International License.

**Conclusion:**

Intraoperative navigation yields higher accuracy and safety for screw placement in the upper cervical spine. Recent improvements of intraoperative cone-beam CT with robotic image-assistance technique provide better image quality in the operation room, allowing surgeons to further minimize surgery-related risks.

**Navigated robot assisted spinal surgery- an evaluation of the first 100 cases**
ID: 120

**Kategorie: DGNC - Intraoperative Bildgebung und Robotik an der Wirbelsäule**

Ersin Haciyakupoglu[1]

[1]*Heinrich-Braun-Klinikum, Standort Zwickau, Neurosurgery, Zwickau, Deutschland*

**Background:**
The robot-assisted systems are increasingly used in operations to improve the accuracy of pedicle screws in spinal surgery. Surgeons can use robotic systems in almost all spinal procedures such as degenerative, fracture, stenosis, scoliosis and at any vertebral level. A 3D or 4D scanning provides real-time information necessary for planning the screw placement and enables the insertion of accurate screws even in the presence of severe degeneration or anatomical variations. Studies on robot-assisted spine surgeries demonstrate an overall accuracy level of 98 percent in these operations.
We would like to present our experience with the Robotic Spine Surgery. We have been using an Operation Robot (Excelsius GPS, Globus Medical) integrated with a 4D Scope (Artis-Pheno, Siemens) in our hybrid operation theatre since 2020. We performed 101 robot-assisted operations in these two years.

**Materials and methods:**
Excelsius GPS (Globus Medical) is a robotic navigation platform which combines a rigid robotic arm and full navigation capabilities in one adaptable platform. Artis Pheno (Simens) allows surgeons to take fast, 4D, high quality scans.
The scans we perform before the operations are automatically sent to the robotic system. We plan the screw location and size on the robotic system and subsequently place the screws with robotic assistance. Following the screw placement, we conduct another 4D scan to detect the accuracy.

**Results:**
We operated on 101 Patients in two years. These operations include the following: 6 Spondylodiscitis (4 Lumbar, 2 Thoracic), 60 fractures (32 Lumbar, 28 Thoracic), 11 metastasis (2 Lumbar, 9 Thoracic) and 24 Stenosis (22 Lumbar, 2 Thoracic) including spondylolisthesis. We had no significant complications in any of these operations. Out of the 792 screws we planted, only two had to be revised (>99%), and this revision procedure was done immediately after the 4D scan detecting the accuracy of the placement. In two years, we improved our robotic system assisted screw implantation time by about 35 percent. We faced wound healing problems in two patients, which we resolved through operations during their hospital stay.

**Conclusion:**

The robotic systems are safe and easy to use and learn. Screw placement accuracy is very high in robotic system assisted surgeries. Especially in patients with severe degeneration or anatomical variations, using such systems is advantageous and safe. Surgeons can use robotic systems in all spinal levels and all kinds of spinal instrumentation procedures. The surgical team's radiation exposure is minimal in surgeries that use robotic systems. Although these systems are expensive, capital recovery takes a short period of time, especially at larger spine clinics. Robotic systems are developing rapidly, getting better and faster. If this rapid development continues, many spinal operations will routinely use robotic systems.

**Robot-assisted navigated pedicle screw placement of the spine**

ID: 148

**Kategorie: DGNC - Intraoperative Bildgebung und Robotik an der Wirbelsäule**

Maximilian Schwendner[1,2], Sebastian Ille[1,2], Raimunde Liang[1,2], Bernhard Meyer[1], Sandro Krieg[1,2]

[1]*Klinikum rechts der Isar, TU München, Klinik und Poliklinik für Neurochirurgie, München, Deutschland*, [2]*TUM Neuroimaging Center TU München, München, Deutschland*

**Background:**

Dorsal instrumentation of the spine using pedicle screws and rods is a standard procedure in spine surgery. Technical advances such as three-dimensional imaging, neuronavigation and robot-assisted systems showed superior results when compared to conventional fluoroscopic screw placement. The aim of this study was to evaluate the clinical implementation of a new system for robot-assisted navigated pedicle screw placement.

**Materials and methods:**

Patients scheduled for pedicle screw placement were prospectively included, as well as an evaluation of the surgeon. Screw placement was performed under assistance of spinal navigation based on intraoperative three-dimensional imaging. Pedicle screw trajectories were adjusted according to intraoperative screw planning using a robot-assisted system. Screw positioning was evaluated according to the Gertzbein and Robins classification (GR). The system was evaluated by the surgeons regarding mental load (scale 0-150).

**Results:**

A total of 15 patients aged 72.3 +/- 16.6 years were prospectively analyzed from june to december 2021. Overall, 108 pedicle screws were implanted. 75 (69.4%) screws were implanted using robotic assistance, 31 screws were placed under CT-guided neuronavigation. In six patients (40.0%) screw placement was performed completely robot-assisted. In nine patients limitations occurred – due to mechanical limitations of the reference array or robotic arm (six cases), increased duration of screw placement (one case) and availability of the robotic arm (one patient). Screw positioning was GR category A and B in 71 screws (94.7%), while intraoperative screw revision was required for one screw (1.3%). Evaluations showed an average mental load of 70 (20-150) for adjustment of the trajectory, 50 (10-140) for installation of the drill guide and 20 (10-150) for drilling of the trajectory.

**Conclusion:**

This new technique of navigated robot-assisted pedicle screw placement is feasible, safe and easily applicable in clinical routine. It enables precise instrumentation of the complete spine and adds up to conventional imaging-based spinal navigation by providing further automation.

**Angulation-Errors in iCT navigated dorsal instrumentation of the spine. An interim analysis of intraoperatively planned trajectories vs. definitive screw placement.**
ID: 200

**Kategorie: DGNC - Intraoperative Bildgebung und Robotik an der Wirbelsäule**

Ulf Bertram[1], Laure Weis[1], Tobias Schmidt[1], Hans Clusmann[1], Christian Blume[1], Christian-Andreas Müller[1]

[1]*Uniklinik RWTH Aachen, Klink für Neurochirurgie, Aachen, Deutschland*

**Background:**
Navigated screw placement is of increasing importance in spinal instrumentation surgery, due to its superior accuracy in contrast to conventional techniques. Especially cervical pedicle screw placement is considered safe only with the use of a navigated approach. However, even intraoperative CT (iCT) navigated pedicle screw placement is prone to error. Recently we have been confronted with the question of whether intraoperatively planned screw trajectories can be considered true with respect to actual screw placement and how angulation errors correlate with screw-misplacement.

**Materials and methods:**
Between March and December 2021 we prospectively recorded intraoperatively planned trajectories of 279 screws in 54 patients placed throughout the spine (104 cervical; 93 thoracic; 76 lumbar; 6 sacral) using BrainLab Curve Planning Station. 3D coordinate systems of planning and control CT scans were merged an vectors of individual screws compared to planed trajectories.

**Results:**
Prior to intraoperative correction of misplaced screws 81% of all screws were placed accurately (<2mm), 14% showed minor (<4mm) and 5% major perforations of the pedicle wall. The mean axial angulation error was 3.4° ± 2.9 and sagittal 3.6° ± 3.7. Rotational (axial) angulation error was higher in the cervical (median 3.4 95%CI 3.6-4.8) then in the thoracic (2.3 95%CI 2.3-3.4) spine (p=0.016). Overall axial and sagittal angulation error correlates positively with increase in Gertzbein&Robbins grade (Spearmans r: 0.19 and 0.23).
Major pedicle wall breaches (>4mm) were found in cases of median sagittal angulation error of 8.3° (95%CI 4.1°-16°) while accurately placed screws featured a median sagittal error of 2.4° (95%CI 2.7°-3.4°). Median axial angulation errors in major misplacements were 4.4° vs. 2.5° regarding acceptably placed screws in our cohort.

**Conclusion:**
iCT remains a solid tool to facilitate accurate dorsal instrumentation throughout the spine. Rotational error of actually achieved compared to intraoperatively planned trajectories is confined to a minimum.

However, angulation error correlates with pedicle violation and cannot be eliminated completely. The specific regional mobility of the spine must be kept in mind when applying pressure on the navigated drill-guide during pediculation to minimize angulation error.

**Intraoperative Computed TomographyeAssisted Spinal Navigation in Dorsal Cervical Instrumentation: A Prospective Study on Accuracy Regarding Different Pathologies and Screw Types**

ID: 201

**Kategorie: DGNC - Intraoperative Bildgebung und Robotik an der Wirbelsäule**

Ulf Bertram[1], Tobias Schmidt[1], Hans Clusmann[1], Walid Albanna[1], Christian Herren[2], Alexander Riabikin[3], Christian Mueller[1], Christian Blume[1]

[1]*Uniklinik RWTH Aachen, Klinik für Neurochirurgie, Aachen, Deutschland*, [2]*Uniklinik RWTH Aachen, Klinik für Unfallchirurgie, Aachen, Deutschland*, [3]*Alfred Krupp Krankenhaus, Klinik für Radiologie und Neuroradiologie, Essen, Deutschland*

**Background:**

Intraoperative computed tomography (iCT) navigated dorsal instrumentation has been success- fully introduced as a new clinical standard. The proximity of vital anatomic structures makes cervical spine instru- mentation an especially delicate task. Therefore, navigated approaches might prove to be beneficial. In this study, the accuracy of conventional instrumentation was compared with iCT navigated dorsal cervical spine instrumentation with focus on cervical pedicle screws (CPSs) versus lateral mass screws (LMSs) and pathologies.

**Materials and methods:**

We analyzed a prospective consecutive se- ries of patients undergoing cervical dorsal instrumentation with iCT and spinal navigation and retrospectively analyzed a cohort that received conventional cervical instrumentation with C-arm fluoroscopy (control group). Accuracy was assessed with a modified Gertzbein-Robbins classification. Underlying pathologies were taken into account regarding accuracy in different entities.

**Results:**

Fifty-nine patients were treated using iCT (357 screws: 238 CPSs, 119 LMSs), and 98 patients underwent conventional instrumentation (632 screws: 69 CPSs, 563 LMSs). We achieved an initial accuracy of 93.28% (n [ 220 screws) in the iCT group and 80.9% (n [ 511 screws) in the control group ($P < 0.001$). Significant differences were found regarding the accuracy of CPS placement in cases of degenerative disorders (iCT vs. control; 94% vs. 63%; $P < 0.001$) and trauma (iCT vs. control; 88% vs. 72%; $P < 0.05$). iCT yielded favorable precision rates in regard to LMS placement (iCT vs. control; 94.2% vs. 82%; $P < 0.05$).

**Conclusion:**

Accuracy of iCT navigated instrumen- tation was significantly higher than conventional instru-mentation. An overall tendency toward the use of CPSs with iCT navigation is evident, increasing the mechanical properties of the construct. iCT appears to be especially beneficial in elective surgery cases of degenerative spinal disorders.

**Minimally invasive 3D-navigated osteosynthesis of fragility fractures of the pelvic ring using intraoperative computed tomography: a single center's experience from four years practice**
ID: 883

**Kategorie: DGNC - Intraoperative Bildgebung und Robotik an der Wirbelsäule**

Andreas Kramer[1,2], Martin Naisan[2], Stefan Kindel[1], Marcus Richter[2], Florian Ringel[1], Philipp Hartung[2]

[1]*Klinik und Poliklinik für Neurochirurgie, Universitätsmedizin, Mainz, Deutschland*,
[2]*Wirbelsäulenzentrum St.-Josefs Hospital, Wiesbaden, Deutschland*

**Background:**
Geriatric patients benefit from the development of minimally invasive techniques with shortened operative times and reduced soft tissue trauma. As a technical evolution of the percutaneous sacroiliac screw fixation (PSS) under fluoroscopic guidance, we here present the 3D-navigated monoportal bilateral PSS of fractures of the pelvic ring and report four years of experience in the application of this technique.

**Materials and methods:**
Records of all consecutive patients that received 3D-navigated monoportal bilateral PSS of fragility fractures of the posterior and/or anterior pelvic ring of 2018-2021 were analyzed. 3D-datasets were acquired using intraoperative computed tomography. Fracture etiology and morphology, pre- and postoperative clinical status and surgical as well as radiographic data of each patient were retrospectively assessed. Fractures were categorized by anatomical configuration and classified using the Fragility Fractures of the pelvis score (FFP). The surgical technique is described and illustrated step-by-step. Aim of this study was to evaluate the feasibility and safety of the investigated technique.

**Results:**
A total of 47 patients - 37 (79%) female, 10 (21%) male - with a median age of 81 years were included in this study. Classification of the fractures resulted in 23 FFP 2 (49 %), 5 FFP 3 (10.5 %) and 19 FFP 4 (40.5 %) fractures. In 36 cases (84 %), only lengthened sacroiliac screws were implanted at the level of S1 and S2 or both screws at S1 or S2, which took a mean incision-to-suture time of 50 min. In 6 cases (13 %) an additional partially threaded screw was implanted to fix a fracture of the anterior pelvic ring resulting in a mean incision-to-suture time of 71 min. Perioperative nerve root, vascular or pelvic organ injury did not occur. Except one patient, who remained bedridden, all patients could be mobilized early postoperatively using a walker or crutches. No wound healing disorders occurred. The median postoperative in-hospital stay was 6 days. During routine follow up examinations, nine patients (19 %) presented with screw loosening, therefore revision surgery was necessary in 4 cases (8.5 %). In multiple regression analysis of potential risk factors for screw loosening, no significant risk factor could be identified.

**Conclusion:**

The presented monoportal bilateral PSS of fragility fractures of the sacrum is a promising minimally invasive technique with a low complication profile and excellent short-term results. Further studies on the biomechanical properties of lengthened sacroiliac screws and comparative analyses with sacroiliac screws inserted biportally are desirable.

### Long-term screw loosening in robot-assisted vs freehand pedicle screw placement: A 10 year single-center retrospective study

ID: 893

**Kategorie: DGNC - Intraoperative Bildgebung und Robotik an der Wirbelsäule**

Maria Elisa DI Francesco[1], Kathrin Machetanz[1], Sasan Adib[1], Florian Roser[2], Marcos Tatagiba[1], Georgios Naros[1]

[1]*Eberhard Karls Universität, Department of Neurosurgery and Neurotechnology, Tübingen, Deutschland*, [2]*Cleveland Clinic Abu Dhabi, Department of Neurosurgery, Abu Dhabi, United Arab Emirates*

**Background:**

There is increasing evidence that robotic assistance improves pedicle screw placement in terms of precision. At the same time, it has been hypothesized that a better pedicle screw placement might prevent long-term screw loosening. However, long-term outcome data is scarce. The present retrospective single-center study aims to evaluate the long-term results between robot-assisted and conventional freehand pedicle screw placement.

**Materials and methods:**

This study enrolls 86 patients undergoing either robot-assisted (ROBOT group, n=60) or conventional freehand (FH group, n=26) pedicle screw placement. We acquired general demographic (e.g., sex, age, body mass index, comorbidities) and surgical parameter (e.g., operative time, number of segments, complications) for both groups. The accuracy of pedicle screw implantation was measured on CT scans based on Gertzbein Robbins grading. Screw loosening rate as well as long-term outcome were assessed by follow-up clinical examination and radiographic imaging.

**Results:**

The groups were equally distributed with regard to demographic parameter (e.g., osteoporosis). Robotic assistance significantly decreased operative time and improved screw positioning. After 7±4 years of follow-up, loosening signs were noted in 12/60 (20%) and 5/26 (19%) for the ROBOT and the FH group, respectively (p=0.832). In contrast, approx. 50% of the patients showed a disease progress with other long-term complications (e.g., subsequent instability, material failure, revision surgery) in both groups.

**Conclusion:**

Robotic assistance improves efficiency and efficacy of pedicle screw placement in spine surgery. Long-term complications (e.g., screw loosening), however, were not affected by the application of the robotic technology but were attributed to patient-specific comorbidities (e.g., osteoporosis).
